# Recombination phenotypes of the NCI-60 collection of human cancer cells

**DOI:** 10.1186/1471-2199-12-23

**Published:** 2011-05-17

**Authors:** Dawn M Stults, Michael W Killen, Brent J Shelton, Andrew J Pierce

**Affiliations:** 1 Department of Toxicology, University of Kentucky, Lexington, KY, USA; 2 Current address: Department of Medicine, Division of Hematology and Oncology, Vanderbilt University, Nashville, TN, USA; 3 Department of Microbiology, Immunology, and Molecular Genetics, University of Kentucky, Lexington, KY, USA; 4 Department of Biostatistics, University of Kentucky, Lexington, KY, USA; 5 Department of Microbiology, Immunology, and Molecular Genetics, Markey Cancer Center, University of Kentucky, Lexington, KY, USA

## Abstract

**Background:**

The NCI-60 is a collection of tumor cell lines derived from a variety of human adult cancer tissue types and is commonly used for genetic analysis and screening of potential chemotherapeutic agents. We wanted to understand the contributions of specific mechanisms of genomic instability to the etiology of cancers represented by the NCI-60.

**Results:**

We screened the NCI-60 for dysregulated homologous recombination by using the gene cluster instability (GCI) assay we pioneered, and for defects in base excision repair by sensitivity to 5-hydroxymethyl-2'-deoxyuridine (hmdUrd). We identified subsets of the NCI-60 lines that either displayed the characteristic molecular signature of GCI or were sensitive to hmdUrd. With the exception of the NCI-H23 lung cancer line, these phenotypes were not found to overlap. None of the lines examined in either subset exhibited significant changes in the frequency of sister chromatid exchanges (SCE), neither did any of the lines in either subset exhibit microsatellite instability (MSI) indicative of defects in DNA mismatch repair.

**Conclusions:**

Gene cluster instability, sensitivity to hmdUrd and sister chromatid exchange are mechanistically distinct phenomena. Genomic instability in the NCI-60 appears to involve only one mechanism of instability for each individual cell line.

## Background

Genomic instability is a fundamental characteristic of most solid tumors and adult leukemias. The term encompasses a broad range of defects that arise by a variety of damaging events and/or mechanistic failures of individual DNA repair pathways. Whatever its source, genomic destabilization is believed to begin early in tumor progression, creating heterogeneity within a population of cells, and conferring, in concert with other events, a selective advantage to a given cell which dominates in proliferation [1,2]. Instability may or may not continue as the tumor progresses. One means of genomic destabilization is defective or dysregulated homologous recombination.

Homologous recombination (HR) is a mechanism for repairing double-strand breaks (DSBs) during S and G2 phase of the cell cycle. In contrast to non-homologous end joining (NHEJ), which results in a loss of genetic material, homologous recombination is considered error-free repair because it uses the available, identical sequence from the sister chromatid to repair the DSB. Although NHEJ is capable of repairing frank DSBs during G2/M, HR is preferred, especially for repairing the DSBs that arise at stalled replication forks, for example from forks that encounter single strand breaks or cross-links [3]. Nevertheless, mitotic HR is a complex, varied, and tightly regulated process, and defects in several of the components of HR have long been associated with cancer (reviewed in [4,5]). One study shows overexpression of several HR-associated genes in patients with non-small cell lung cancer [6]. Approximately 5% of the human genome is comprised of large repetitive elements called low copy repeats (LCRs), also known as segmental duplications, which possess sufficiently high sequence identity to cause structural genomic instability via non-allelic homologous recombination (NAHR) between regions of identical sequence but differing genomic context, resulting in insertions, deletions, and translocations ([7], reviewed in [8]).

The most established means of detecting dysregulated homologous recombination, whether in cells with defective/deficient HR capacity, or in response to damage, is the sister chromatid exchange assay (SCE) that differentially stains sister chromatids, allowing for microscopic detection of the physical exchange of DNA which occurs with crossover HR [9]. With the advent of straightforward techniques, the SCE assay has been in popular use since the 1970s for the purpose of identifying potential "chromosomal mutagenicity" of chemical agents [10]. Chemicals that generate cross-linking of DNA are potent inducers of SCE, since HR is required to repair the resultant blockage during replication [11]. Conditions and drugs which increase the number of single-strand breaks (SSBs) also increase the number of SCEs, presumably by overburdening the base-excision repair (BER) pathway such that unrepaired SSBs remain, become DSBs during replication, and must be repaired by homologous recombination [9]. Accordingly, HeLa cells with downregulated XRCC1, a key component in the base excision repair pathway, show a 1.7-fold increase in SCEs, and an almost 2-fold increase when methyl methansulfonate (MMS), a DNA methylating agent, is added [12]. Likewise, the thymidine analog 5-hydroxymethyl-2'-deoxyuridine (hmdUrd) at a 1 μM dose induces sister chromatid exchanges resulting in a 6-fold increase over background in Chinese hamster ovary (CHO) cells [13], again presumably through either saturation of BER activity, or through DNA replication across nicked BER intermediates. Inhibition or deficiency of poly(ADP-ribose) polymerase (PARP-1) also increases levels of sister chromatid exchange [14]. Essentially, the HR pathway compensates, at least partially, for the defects or inadequacy of the BER response. PARP inhibitors can induce synthetic lethality in cells with mutations in BRCA-1 or BRCA-2, which are components of the HR pathway [15,16]. Similarly, exposure to hmdUrd (or the related compound 5-chloro-2'-deoxyuridine) is synthetically lethal with loss of key BER components such as XRCC1 [17].

Despite the striking visible result upon staining, sister chromatid exchange is genetically silent. It represents a very large scale physical relocation of genetic material which is the consequence of a crossover recombination event; but there is no gain or loss of genetic information between two identical sisters. Presumably, these crossovers happen at the submicroscopic level as well. Our lab has developed an assay which measures non-silent, NAHR-mediated molecular level changes to genomic architecture by monitoring the stability of the length of gene clusters or tandemly repeated segmental duplications [18]. For this gene cluster instability (GCI) assay, we use the gene clusters that produce the 45S precursor transcript to the 18S, 5.8S and 28S ribosomal RNA molecules. These clusters of tandemly repeated genes are located on the small arms of the five acrocentric chromosome pairs, representing a total of approximately 600 copies of the 43kb unit gene [19]. We characterized the lengths of these rDNA gene clusters from healthy blood donors and found complete heterozygosity on each of the five chromosomes, and between the parental pairs of homologs. We also detected abundant evidence of both human meiotic [20] and mitotic rearrangement [18]. We recently used the GCI assay to compare matched normal tissue to tumor tissue in patients with lung or colorectal cancer and found that approximately 50% of the tumors show changes in the sizes of the clusters compared to the normal tissue, as well as evidence of ongoing instability and heterogeneity within the tumor population indicating that HR has at some point become dysregulated within the tumor cells [21]. Notably, loss or knockdown of the RecQ homolog defective in Bloom syndrome (BLM) causes a remarkable 100x increase in rDNA gene cluster instability rates along with the well-characterized 10-fold elevation in rates of sister chromatid exchange in these cells [22], suggesting elevated HR with crossing-over as the most likely mechanism [23] for this destabilization. We also demonstrated that loss of the ataxia-telangiectasia-mutated (ATM) protein causes a 10x elevation in rDNA gene cluster instability, even though loss of ATM in the absence of exogenous DNA damaging agents does not increase levels of sister chromatid exchange [24-26].

We are interested in the manner by which elevated and/or dysregulated recombination may be involved in the etiology of cancer and the development of chemotherapeutic resistance. We reasoned that elevated recombination could be caused either by an increase in recombination initiating lesions as the result of BER deficiency as seen in XRCC1 mutants, or by alterations in the downstream biochemistry of recombination causing an increase crossover vs. non-crossover recombination as seen in BLM mutants. Accordingly, we screened the NCI-60 panel of human cancer cell lines for defective BER by sensitivity to hmdUrd, and for altered recombination outcomes by the gene cluster instability assay. Lines exhibiting either phenotype were subsequently characterized by sister chromatid exchange in order to cross-compare three potential mitotic recombination indicators.

## Results and Discussion

### Gene Cluster Instability Survey

**Figure 1:**
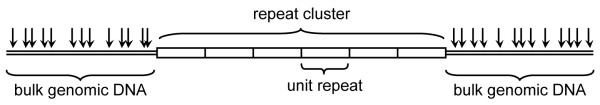
**Gene cluster instability experimental strategy**. Digestion of genomic DNA with restriction enzymes that do not cut within an individual gene cluster repeat liberates intact gene clusters from bulk genomic DNA. Panel from [20]. Vertical arrows: putative restriction enzyme digestion sites. Open rectangles: unit gene cluster repeats.

**Figure 2:**
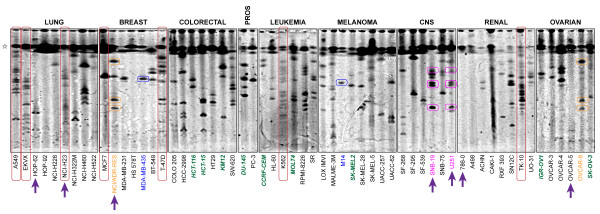
**Screening the NCI-60 for gene cluster instability (GCI)**. The presence of a large number of clusters or variable intensity of clusters suggests instability. Open star: gel resolution limit (1 Mb). Large red rounded rectangles: lines exhibiting gene cluster instability selected for further analysis. Small rounded rectangles: matching bands consistent with multiple lines derived from the identical patients. Known paired lines: NCI/ADR-RES & OVCAR-8 (orange), MDA-MB-435 & M14 (blue), SNB-19 & U251 (mauve). Purple arrows: lines sensitive to hmdUrd. Green italicized names: lines exhibiting microsatellite instability and defects in DNA mismatch repair.

For these experiments, we used our gene cluster instability (GCI) assay to measure dysregulated recombination by identifying changes in the lengths of ribosomal RNA gene (rDNA) clusters. High molecular weight genomic DNA was digested with EcoRV (New England Biolabs), which cuts the human genome into fragments with an average length of 6600 base pairs [27], but does not have a recognition site within the single rDNA repeat. The sequence of these repeats is highly conserved, and thus, an enzyme which does not cut within a single repeat generally does not cut anywhere in the cluster of tandemly repeated genes, and the entire cluster can be separated from rest of the genomic DNA by pulsed-field gel electrophoresis, and identified by Southern blotting (Figure 1). We have found that a pulsed-field gel with a resolution limit of 1MB is the most informative for tracking changes in cluster length associated with GCI. We used this approach to screen the entire NCI-60 panel of 59 cancer cell lines to identify those which showed evidence of rDNA cluster instability (Figure 2). We have previously shown that Bloom syndrome cells, which demonstrate a ladder-like banding pattern, are highly unstable on GCI analysis [18]. It was on this basis that we identified six lines which demonstrated laddering indicative of instability as candidates for further analysis. Three of these were lung cancer lines (A549, EKVX, and NCI-H23), one was leukemia (K562), one was breast cancer (T47D), and one was renal cancer (TK10). According to our previous findings in tumor versus non-tumor tissue from the same patient, the frequency of rDNA cluster instability is about 50%, as indicated by any variation in banding pattern between the normal and malignant tissue at the 1 MB resolution limit [21]. For these experiments, we did not have access to normal tissue for comparison, and it is likely that many more than the six lines we chose show differences from matched normal tissue. Central nervous system (CNS) cell lines SNB19 and U251 were derived from the same patient. According to our GCI results, they demonstrate a similar pattern, with four common bands, but also deviate from one another with a total of five bands that are not shared, indicating that the rDNA has continued to undergo rearrangement in culture. NCI/ADR-RES and OVCAR8 are also derived from the same patient, but NCI/ADR-RES has acquired adriamycin resistance through escalating challenge with this drug. These lines share three common bands, but NCI/ADR-RES appears to have acquired an additional band in culture. The fact that these two lines demonstrated so little divergence in culture despite the adriamycin-mediated DNA damage and acquired resistance indicates likely mechanistic specificity to the type of DNA damage that can initiate gene cluster instability. A third cell line pair, MDA-MB-435 and M14, are also derived from the same donor. However, these lines have only a single band below 1MB and it appears stable. Nine of the NCI-60 lines (HCT-116, HCT-15, KM12, DU-145, CCRF-CEM, MOLT4, SK-MEL2, IGR-OV1, SK-OV-3) are known to demonstrate microsatellite instability indicative of mismatch repair defects [28]. It is notable that none of the mismatch-repair deficient lines showed overt evidence of dysregulated recombination on the initial GCI screen. Although genomic instability is a hallmark of cancer, it is possible that a tumor cell may only need a single means of acquiring instability to confer a selective advantage; and thus mismatch repair defects and recombination defects are mutually exclusive within a given tumor cell.

### Lines with ongoing GCI: TK-10, K562, T-47D

**Figure 3:**
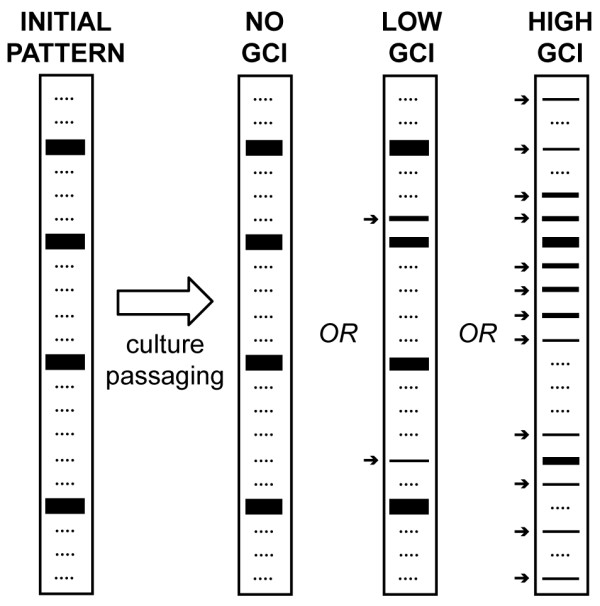
**Schematic of subclonal assay to measure GCI rate**. A single cell shows a well-defined pattern of gene cluster lengths ('Initial Pattern'--thick bands). Allowable, but currently unrepresented gene cluster lengths are shown as dotted lines. As mitotic division expands the clonal cell population in the absence of instability, gene cluster lengths are faithfully preserved ('No GCI'). Alternatively, instability generates sub-populations within the expanding population with altered cluster lengths giving rise to lower intensity 'minor bands' ('Low GCI'--thin bands indicated by arrows). High levels of instability generate a ladder-like pattern of minor banding with individual bands on the ladder differing by integer multiples of the unit repeat length ('High GCI'). Adapted from [18].

For the six lines that showed laddering similar to that seen in Bloom syndrome (Figure 2, circled in red), we undertook a subclone analysis to determine whether dysregulated recombination and subsequent instability was an ongoing process. We duplicated our approach from the previous Bloom syndrome experiments [18]. We began by isolating colonies derived from a single cell from the populations that demonstrated gene cluster laddering. In general, the parental population derived from a single cell usually demonstrates an initial, well-defined "major banding pattern". Colonies from single cells from the cloned parental plate were grown and GCI analysis was performed. As each clonal population expands, if the rDNA clusters are completely stable, the initial cluster lengths found in the parental line will be faithfully transmitted to all subsequent daughter cells (Figure 3, 'No GCI'). Alternatively, recombination in the expanding population can generate sub-populations with altered gene cluster lengths. Since these sub-populations only represent a fraction of the total population, bands will be reduced in intensity accordingly. We call these reduced-intensity bands the "minor banding pattern" (Figure 3, 'Low GCI') since the intensity of these bands is non-stoichiometric with respect to the length of the cluster detected. The amount of this minor-intensity banding found in any cell population is indicative of the degree of GCI in that population. Since recombination requires precise alignment of homologous sequences, cluster lengths can only change by integer multiples of the unit repeat length. This constraint upon allowable gene cluster lengths (Figure 3, dotted lines) means that very high levels of instability will generate a ladder-like pattern of bands (Figure 3, 'High GCI'), consistent with a recombination-based mechanism. If cluster length alterations were due to random breakage and rejoining, a smear would be observed, rather than a ladder [18]. As in the case of Bloom syndrome, lines which demonstrated a laddering pattern in the population sometimes showed non-stoichiometric bands within the parental population, indicating a high degree of instability even within the first parental expansion.

**Figure 4:**
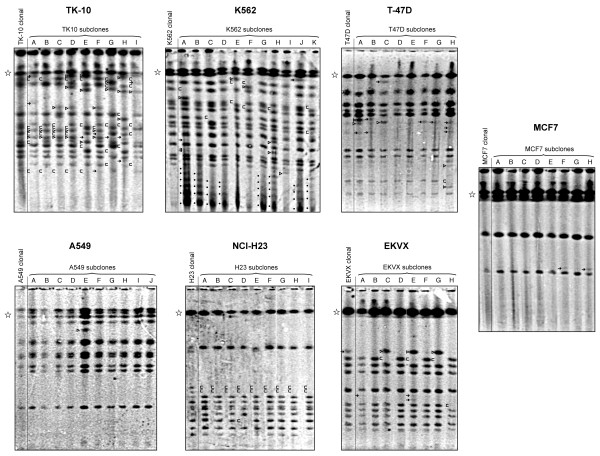
**Clonal GCI assays**. for each cell line analyzed, TK-10, K562, T-47D, A549, NCI-H23, EKVX and MCF7, clonal populations are on the far left of each panel with individual subclones derived from single cells in the clonal population on the right. Open star: 1 Mb resolution limit. Brackets: clusters missing in the subclones, yet present in the clonal population. Open triangles: clusters found in the subclones, yet missing in the clonal population. Arrows: minor intensity bands from sub-populations arising from mitotic recombination during early subclonal expansion. Low molecular weight dotted pattern: cluster fragments derived from site-specific fragility in the cluster broken during DNA processing.

We characterize the renal cell cancer TK-10 as having high GCI (Figure 4). Among the nine subclones there were a total of nine new minor bands (black arrows), 24 major bands either new or inherited from minor bands in the parental population (open triangles), and 35 deletions of either major or minor bands from the parental population (brackets). K562 also showed high GCI (Figure 4), with three new minor bands (black arrows), six new major bands (open triangles), and nine major band deletions (brackets) among the 11 subclones. Interestingly, K562 subclones also showed a stoichiometric laddering pattern of bands which differ by a length of a single unit repeat (black dots) in the lower range of the gel. We do not believe these arose by dysregulated homologous recombination, but rather through site-specific fragility in the rDNA unit repeat during DNA isolation. Although we did not observe this phenomenon in any of the other subcloned lines, it does sometimes develop in solid tumor and non-tumor tissue [21]. T-47D also appeared unstable on subclone assay (Figure 4), with the appearance of eight new minor bands (black arrows), five new major bands (open triangles), and one major band deletion (bracket) across eight subcloned lines. To contrast with a line exhibiting gene cluster stability, we also expanded a parental clone and conducted subclone analysis for MCF7, a breast cancer line that is well characterized and frequently used for molecular biology experiments (Figure 4). MCF7 did not demonstrate laddering on the initial screen (Figure 2) and showed only two major bands below the 1MB resolution limit of the gel. MCF7 subclones showed two new minor bands (black arrows) and no new major bands or deletions among the eight subclones.

In our previous work with ATM and BLM deficient cells [Killen, 2009 #1052], we estimated GCI rates in terms of observed new minor intensity bands per subclone. To a first approximation, wild-type cells had a GCI rate of 0.1 minor bands per subclone compared to rates of 1.0 and 10 minor bands per subclone in ATM and BLM deficient cells respectively. By this measure, the rates of instability in TK-10, K562, T-47D cells are more qualitatively similar to the 10x elevated rate in ATM-deficient cells over wild-type rates, rather than the more extreme 100x elevated rate in BLM-null cells.

### Lines with historical GCI, but currently stable: A549, NCI-H23, EKVX

Lung cancer cell line A549 does not appear to demonstrate ongoing rDNA cluster instability (Figure 4). The major banding pattern from the clonal parental line is faithfully transmitted to each of the daughter lines with the exception of a single additional major band in subclone E (Figure 4, open triangle). Lung cancer line NCI-H23 shows similar results, with the disappearance of a single band in subclone D (Figure 4). All nine subclones also appear to have lost two bands that are non-stoichiometric but apparent in the parental line. We attribute this change to the technical details of culturing this specific line: NCI-H23 does not form single-cell-derived colonies easily, cells tend to migrate toward one another, and it may be that the parental population was not purely derived from expansion of a single cell but rather became mixed with an independent clone during the expansion process. We classify the third lung cancer cell line showing cluster length laddering, EKVX, as having low GCI on the basis of subclonal analysis (Figure 4). The parental line shows a single non-stoichiometric band thought to have arisen during expansion by mitotic recombination. Three of the subclonal lines appear to have inherited this band (subclones C, E, and G), while the other five (A, B, D, F, and H) did not.

### Lack of overlap between GCI and defective mismatch repair

Nine of the NCI-60 collection of human tumor lines have defects in mismatch repair with resulting microsatellite instability (Figure 2). The lack of overlap between lines with gene cluster instability and mismatch repair defective lines emphasizes the mechanistic differences between these two aspects of genomic instability.

### Sensitivity to hydroxymethyl deoxyuridine (hmdUrd): HOP-62, NCI-H23, SNB-19, U251, OVCAR-5, NCI/ADR-RES, 786-0

**Figure 5:**
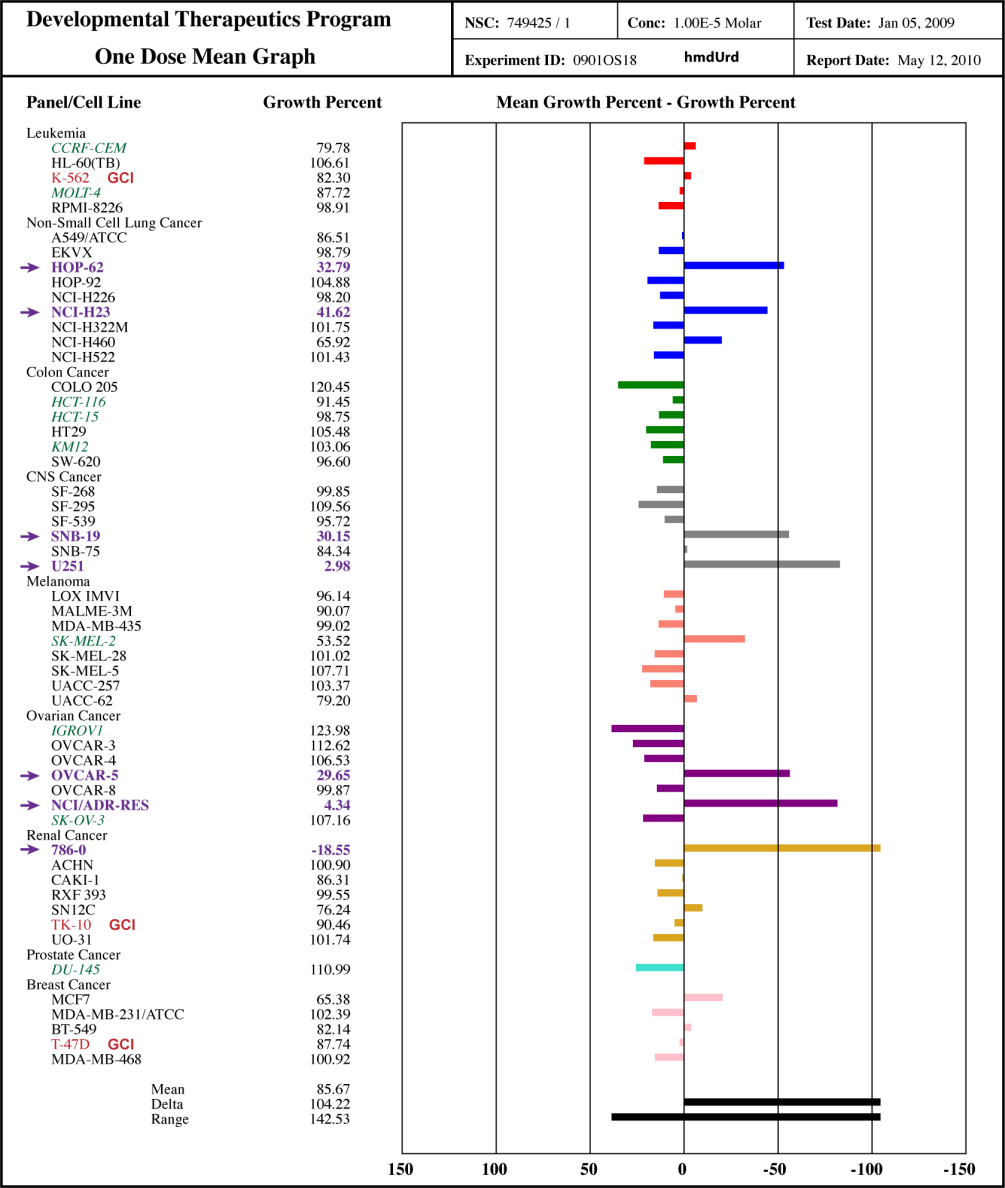
**5-hydroxymethyl-2'-deoxyuridine (hmdUrd) sensitivity**. Sensitivity screen for a single 10 μM dose. Red text: lines with ongoing gene cluster instability seen in clonal analysis. Purple text & arrows: lines sensitive to hmdUrd. Green italicized text: lines with microsatellite instability from defects in DNA mismatch repair.

As a functional screen to identify lines exhibiting hyper-recombination as a result of defective BER biochemistry, we looked for sensitivity to killing with hmdUrd. The lines HOP-62, NCI-H23, SNB-19, U251, OVCAR-5, NCI/ADR-RES and 786-0 showed good sensitivity to growth inhibition in a single dose growth inhibition assay (Figure 5). The lines we identified as exhibiting ongoing gene cluster instability, TK-10, K562 and T-47D did not show hmdUrd sensitivity, suggesting that the GCI phenotype of these lines is not related to defects in base excision repair. It is particularly interesting that the NCI/ADR-RES line is sensitive to hmdUrd, while the OVCAR-8 line is insensitive. These lines are derived from the same patient (Figure 2 and [29]), with the NCI/ADR-RES line selected for growth in progressively higher concentrations of adriamycin [30]. It seems likely that the OVCAR-8 line became compromised in BER due to genotoxic damage during adriamycin selection. It must be noted, however, that we did not directly measure the incorporation of hmdUrd into the DNA of treated cell lines. HeLa cells, for example, are known to be insensitive to hmdUrd due to a failure to incorporate the molecule into DNA [31]. If similar resistance mechanisms apply to the NCI-60 cells, there may yet be BER deficient lines in the NCI-60 collection that are not identified by the hmdUrd sensitivity screen.

### Sister Chromatid Exchange

**Figure 6:**
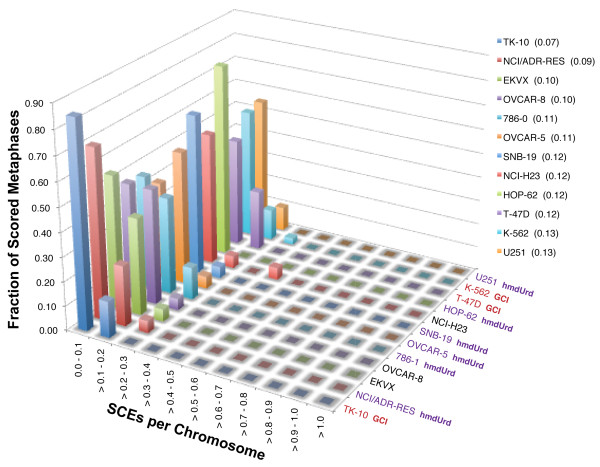
**Sister chromatid exchange distributions in select NCI-60 lines**. Lines are arranged from fewest median SCEs per chromosome (TK-10) through highest median SCEs per chromosome (U251). Red text: lines with ongoing gene cluster instability seen in clonal analysis. Purple text: lines sensitive to hmdUrd.

We performed sister chromatid exchange analysis on five of the lines which showed initial and/or ongoing instability (EKVX, NCI-H23, T-47D, TK-10, and K562) (Figure 6), in order to explore whether elevated levels of gene cluster instability correlate with elevated levels of sister chromatid exchange, as is the case for cells defective in the Bloom syndrome protein BLM [18]. There appears to be some variability between the lines scored, but in general the number of exchanges is around 0.125 per chromosome, consistent with values from wild type fibroblasts (GM00637) that are stable by the GCI assay [18].

Comparison of the two lines showing the greatest difference in median SCE/chromosome values, TK-10 (0.07) and U251 (0.13) indicates that the difference between these two lines is statistically significant (P = 0.017, Bonferroni corrected Mann-Whitney test). Nevertheless, we observed that, although the median number of chromosomes per metaphase in TK-10 is not out of line with values for the other lines, the chromosomes in TK-10 tended to be shorter on average than the other lines, which may limit our ability to score SCEs near the ends of these shorter chromosomes and artifactually reduce the TK-10 SCE values. A statistically significant difference involving pairwise comparisons between the other lines scored is not seen.

### Levels of BLM Protein in Lines With Ongoing GCI

**Figure 7:**
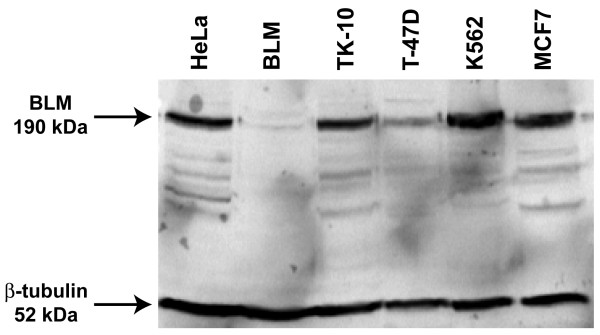
**Western blot for BLM protein in select lines**. Whole cell protein lysates from HeLa, BLM-null (Coriell: GM0857), TK-10, T-47D, K562 and MCF7 cells were blotted. Bands detected with a BLM antibody and with a β-tubulin antibody as a protein loading control are indicated.

We directly assayed the amount of BLM protein in the TK-10, T-47D and K562 lines that demonstrate ongoing GCI (Figure 4), as well as in GCI-stable HeLa and MCF7 cells, along with a known BLM defective line as a control (Figure 7). The T-47D line shows greatly reduced BLM levels relative to the other BLM-containing cells, although T-47D does not display the characteristic increase in SCE seen in BLM-null lines. Accordingly, if BLM depletion is the mechanism of ongoing GCI in T-47D, then elevated GCI would be a more sensitive readout for BLM depletion than would be elevated SCE, although the TK-10 and K562 cells clearly demonstrate that BLM protein level independent mechanisms for GCI must also exist.

## Conclusions

We were surprised to find that gene cluster instability in the NCI-60 panel of cells did not correspond with markedly elevated sister chromatid exchange. This observation establishes clear mechanistic differences between these two processes. Likewise, our observation that hmdUrd sensitivity in the NCI-60 panel of human cell lines did not correspond well to elevated SCE activity previously demonstrated in CHO cells may point to differences in physiology and metabolism between humans and rodents.

In summary, of the six lines we identified as definitively demonstrating rDNA GCI instability on initial screen, only three (T47D, TK10, and K562) continue to show evidence of ongoing instability at the present time. The other three appear to have accumulated gene cluster changes in the process of establishing and culturing the cell lines as the result of transient exogenous influences, rather than due to an endogenous mechanistic switch to an elevated gene cluster instability phenotype. These results are in good accord with our previous observation in gene cluster unstable human solid tumors [21] that half of the cases could be attributed to historical genotoxic damage, while half were more suggestive of a biochemical alteration towards an ongoing predisposition to gene cluster instability.

## Methods

### Cell Culture

NCI-60 cell lines were acquired frozen from the National Cancer Institute's Developmental Therapeutics Program (DTP), and grown in RPMI-1640 culture medium supplemented with antibiotics and 5% fetal bovine serum. Cells were maintained in this medium, at 37C, in 5% CO_2_, in a humidified incubator for the duration of these experiments.

For subclonal GCI analysis, clonal cultures were initially generated from the bulk NCI-60 cell populations as received from the DTP by isolating single cells through limiting dilution followed by unrestricted expansion. For generating multiple subclonal cultures, single cells from the freely expanding clonal cultures were again isolated by limiting dilution followed by unrestricted expansion.

### High Molecular Weight DNA Isolation

Single cell suspensions of 1x10^7 ^cells/mL in 0.8% low melting point were drawn into a 1-mL syringe, and chilled on ice until solidified. High molecular weight DNA was prepared from the solid-phase agarose cell suspension by digestion with 1% sarkosyl/500 mM EDTA/0.5 mg/mL proteinase K solution at 50C for at least 16 h, after which it was treated with phenylmethylsulfonyl fluoride in 10 mM Tris/1 mM EDTA (TE pH 8.0), extensively rinsed in TE/50% glycerol, and stored at -20C.

### Gene Cluster Instability Analysis

The rRNA gene clusters were analyzed by pulsed-field gel electrophoresis and Southern blotting essentially as described [20]. Briefly, approximately 1 μg of genomic DNA in solid phase was digested with EcoRV (New England Biolabs) overnight to release intact gene clusters from bulk genomic DNA. Digested DNA was placed into wells of a 1% Pulse Field Certified (Bio-Rad) agarose gel constituted in 0.5x TBE (44.5 mM Tris/44.5 mM boric acid/1.0 mM EDTA pH 8.0) and samples sealed into the wells using 0.8% low-melting-point agarose. Gels were run using a CHEF-MAPPER (Bio-Rad) at 14C, with two 6V/cm field vectors at 120° separation, with a switch time of from 3" to 90" over 24 hours using a 0.357 ramp factor. Following electrophoresis, gels were equilibrated to a final concentration of 0.5% glycerol in water and dried at 65C. Dried gels were rehydrated and the DNA denatured using 0.4 N NaOH/0.8 mM NaCl, neutralized in 0.5 M Tris pH 8.0/0.8 mM NaCl, then prehybridized at 65C in a hybridization buffer of 2x SSC (300 mM NaCl/30 mM Na-citrate) with 7% SDS and 0.5% casein. Gels were probed in fresh hybridization buffer with an rDNA-specific, ^32^P-labeled probe (radiolabeled PCR products amplified from a plasmid containing cloned human rDNA sequence using primers 5'-GGGCTCGAGATTTGGGACGTCAGCTTCTG and 5'-GGGTCTAGAGTGCTCCCTTCCTCTGTGAG) at 65C overnight. Southern probing in rehydrated dried gels maintains quantitative hybridization signal strength while avoiding documented difficulties involved in transferring DNA from pulsed-field gels onto membranes [32,33]. Following two rinses in 2x SSC/1% SDS solution and two rinses in 0.5x SSC/1% SDS, the gels were imaged using a PhosphorImager (Molecular Dynamics).

### Drug Sensitivity Screen

The in vitro growth inhibition screen of NCI-60 screen with a single 10 μM dose of 5-hydroxymethyl-2'-deoxyuridine (hmdUrd) was performed by the National Cancer Institute's Developmental Therapeutics Program according to the methods described online (http://dtp.nci.nih.gov/branches/btb/ivclsp.html). Briefly, cells are replica-plated in 96-well microtiter plates. After growth for 24 hours, time-zero plates are fixed with trichloroacetic acid (TCA) while experimental plates are treated with drug or left as untreated controls. Experimental plates are incubated for growth for an additional 48 hours before TCA fixation. Fixed cells are quantified spectrophotometrically by staining with sulforhodamine B and growth values are calculated as the mean of duplicate experiments.

### Sister Chromatid Exchange Analysis

Sister chromatid exchanges were prepared using BrdU and visualized essentially as described [34]. Individual metaphase spreads were photographed using bright-field microscopy and a 60x oil immersion objective.

### Western Blotting

Protein extracts were prepared using RIPA buffer as described previously [18]. Following SDS-PAGE, gels were blotted onto Hybond-ECL nitrocellulose membrane (Amersham Biosciences). Proteins were detected using rabbit antibodies to BLM (Cell Signaling Technologies) and β-tubulin (NeoMarkers) with a horseradish peroxidase conjugated donkey anti-rabbit secondary antibody (Pierce) and an ECL Plus western blotting detection system (GE Healthcare).

## Authors' contributions

DMS performed the majority of the experiments and wrote the manuscript. MWK helped with experimental support and contributed to discussions of the work. BJS contributed statistical support. AJP initiated the work and wrote the manuscript. All authors read and approved the final manuscript.
